# Membrane Fouling Mechanisms in Combined Microfiltration-Coagulation of Algal Rich Water Applying Ceramic Membranes

**DOI:** 10.3390/membranes9020033

**Published:** 2019-02-22

**Authors:** Kitae Park, Pooreum Kim, Hyoung Gun Kim, JiHoon Kim

**Affiliations:** 1 Graduate School of Water Resources, Sungkyunkwan University, Suwon 16419, Korea; poorerm@naver.com; 2 POSCO E&C tower, 241, Incheon tower-daero, Yeonsu-gu, Incheon 22009, Korea; khgun@poscoenc.com

**Keywords:** algal-rich water, ceramic membrane, coagulation, membrane fouling, specific cake resistance

## Abstract

In this paper, we investigated the membrane fouling mechanism according to the coagulant dosage in algal rich water using a ceramic membrane. The algae that were used in this experiment were *Microcystis* sp. of cyanobacteria, and the fouling mechanism was analyzed through irrigation and filtration resistance through a constant flow operation. The experimental results showed that the filtration resistance decreased as the coagulant dosage increased, but the irreversibility at above optimum coagulant dosage increased. Additionally, as the coagulant dosage increased, the resistance value due to cake and adsorption contamination decreased, and membrane fouling by adsorption was dominant in comparison with cake fouling and adsorption fouling. The specific cake resistance was decreased as the coagulant dosage increased. The characteristics of the cake layer according to the coagulant dosage were found to loosely form the cake layer by increasing micro-size algae as the coagulant dosage increased. The results of this experiment confirmed the membrane fouling mechanism according to coagulant dosage when the ceramic membrane filtered algal rich water.

## 1. Introduction

It is expected that global climate changes due to global warming will bring environmental changes that are different from the past. In particular, there is concern that a large outbreak of Cyanobacteria will be prolonged by an increase in water temperature during summer in water environments, such as rivers or lakes. In addition, the occurrence of algal blooms causes serious problems in conventional water treatment processes, including coagulation, flotation, filtration, and disinfection and then eventually causes undrinkable water. Recently, low pressure membrane filtration, such as ultra filtration (UF) and micro filtration (MF), has attracted increasing attention from algal rich water treatment areas because of the higher production of treated water and the lower operating costs. According to preliminary studies [1,2,3], a membrane process was able to completely remove algal cells, and a hybrid process combined with activated carbon was able to remove extracellular organic matter (EOM). However, membrane fouling remains an important issue until membrane technologies are widely applied. It has been reported that algal cells, including similar substances, such as polysaccharides, proteins, lipids, and humus mainly cause membrane fouling of algae-rich water [4]. The forms of membrane fouling include adsorption or gelation between algal particles and the membrane surface and formation of pore blockage or cake layers [5].

Numerous control strategies were proposed for mitigating the fouling resistances of membrane: feed pretreatment (coagulation, adsorption), change of feed characteristics (temperature, pH, dissolved oxygen), membrane modification (grafting, coating), optimizing operational parameters (cross-flow), hydraulic flushing, two-phase flow scouring, enhanced shearing, enhanced field (electric, ultrasonic, magnetic [6]), and concentration polarization drawer [7]. Recently, the process combined with coagulation, adsorption, and oxidation processes have been widely studied to control membrane fouling [8]. In particular, it was reported that coagulation pretreatment was an effective alternative for improving membrane performance [9].

It has been reported that coagulation is the most common process in water treatment and it plays an important role in removing not only NOM (Natural organic matter), but also EOM [10]. Coagulation conditions such as type, dose, and mix mode of coagulants have a large effect on coagulation-membrane processes [10,11]. Konieczny et al. [12] reported that the aluminum salt coagulant showed higher removal efficiency than ferric salt.

The purpose of this paper is to analyze membrane fouling according to the injection rate of Polyaluminum chloride (PaCl) as an aluminum coagulant by analyzing the raw water of the lake where algae occurred. Accordingly, this study was performed to examine membrane fouling according to the injection rate of PaCl and to understand the cake properties of algae particles that are attached to the membrane surface under various coagulation conditions. To this end, this study examined the fouling mechanism by analyzing the filtration resistance according to the injection rate of PaCl and analyzed the cake behavior using existing cake resistance models.

## 2. Materials and Methods

### 2.1. Charateristics of the Raw Water

The experiments were performed using raw water that was sampled from Daecheongho Lake located in Cheongju-si, Chungcheongbuk-do, South Korea. This study analyzed the cell density and community species of algae to analyze the algae of raw water. As a result, the community index of *Cyanobacteria Microcystis sp.* was 0.999. Table 1 shows the detailed results.

### 2.2. Experimental Set-Up

All of the experiments were conducted using laboratory-scale membrane set-up, which mainly included a raw water tank, a constant level water tank, a peristaltic pump, a pressure transducer, and a data acquisition system. Table 2 shows the specifications of the membranes used in this experiment. The immersed flat type MF membrane module (Cembrane, Denmark) was made of silicon carbide (SIC), with an effective membrane area of 0.0652 m^2^ and a nominal pore size of 0.1 µm. The reactor (effective volume of 16.4 L) was fed with raw solution through the constant level tank and the effluent was directly drawn from the membrane module by the peristaltic pump (EMS-2000S, Korea). A pressure transducer (PTP708 Tuopo Electric, Korea), which was connected to a laptop computer, was used to continuously monitor the transmembrane pressure (TMP).

### 2.3. Operating Conditions

All of the ceramic membrane fluxes were maintained at 60 LMH, and the reactor temperature was adjusted to 20 ± 0.5 °C using a water bath. Air scrubbing was able to be operated stably at 1 LMH through preliminary experiments. The experiments were performed by applying it. Prior to the use, each membrane was washed and then flushed using ultrapure water under the same conditions. The coagulation conditions were adjusted to pH 7.5 ± 0.3 with 0.01 N H_2_SO_4_ and 0.01 N NaOH.

### 2.4. Measurement of Resistance

Membrane fouling due to algal deposition was studied by measuring the following equations at a constant permeate flux (60 LMH) and water temperature (20 ± 0.5 °C). In the filtration experiments, the specific membrane resistance was first measured using ultrapure water.

$$R_{c}=\frac{\Delta P}{\mu J}-R_{m}$$

where
*R_c_*the resistance of algal cake (1/m)ΔPthe transmembrane pressure (Pa)*J*the filtration flux (m^3^/m^2^/s)*R_m_*the resistance of membrane (1/m)


*R_c_* can be made from the perspective of specific cake resistance, as follows:

Furthermore, α is directly affected by the cake pressure gradient ∆P, and the functions of α and ∆P take the following forms [13,14,15,16,17]:

α = α_0_(∆P)^n^

α_0_Empirical constantncake compressibility factor
n has values between 0 and 1. It means non-compressibility as it approaches 0 and compressibility as it approaches 1 [18].

The reversibility and irreversibility according to the coagulant injection rate were analyzed while using the following methods. Membrane fouling was calculated by the following equations in defining the filtration resistance that was obtained by filtering ultrapure water through the initial membrane, the final filtration resistance of the fouled membrane by filtering using the target raw water to be treated, and the filtration resistance that was obtained by filtering using ultrapure water after physical cleaning as R_0_, R_1_, and R_2_, respectively:

$$RF=\frac{R_{2}-R_{1}}{R_{0}-R_{1}}$$

$$IF=\frac{R_{0}-R_{2}}{R_{0}-R_{1}}$$

*TF* = *RF* + *IF* = 1

*RF* means reversible fouling, *IF* means irreversible fouling, and the total membrane (*TF*) becomes 1.

### 2.5. Measure of EPS

The extraction of EPS (Extracellular polymeric substances) was performed by the thermal extraction method in this study [19]. Protein analysis was measured by Bradford Assay method and polysaccharide was measured by the phenol sulfate method. All of the above analyses were conducted in duplicate, and their average values were reported.

## 3. Results and Discussion

### 3.1. Flux Curves and Reversibility Analyses

**Figure 1:**
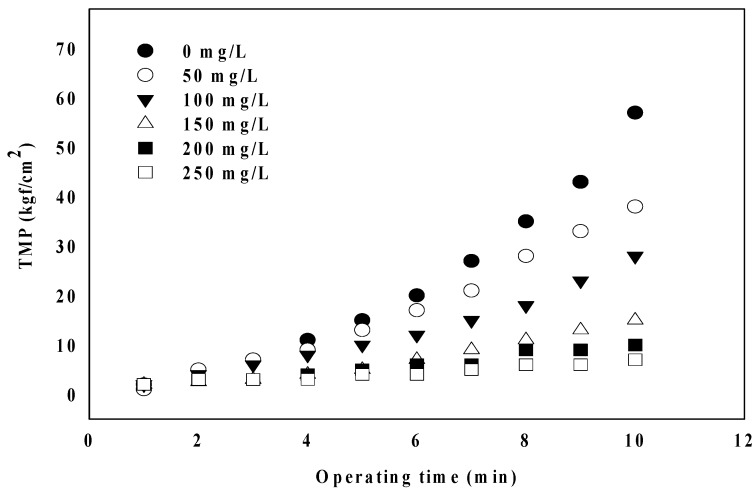
Comparison of changes in transmembrane pressure (TMP) according to coagulant injection.

**Figure 2:**
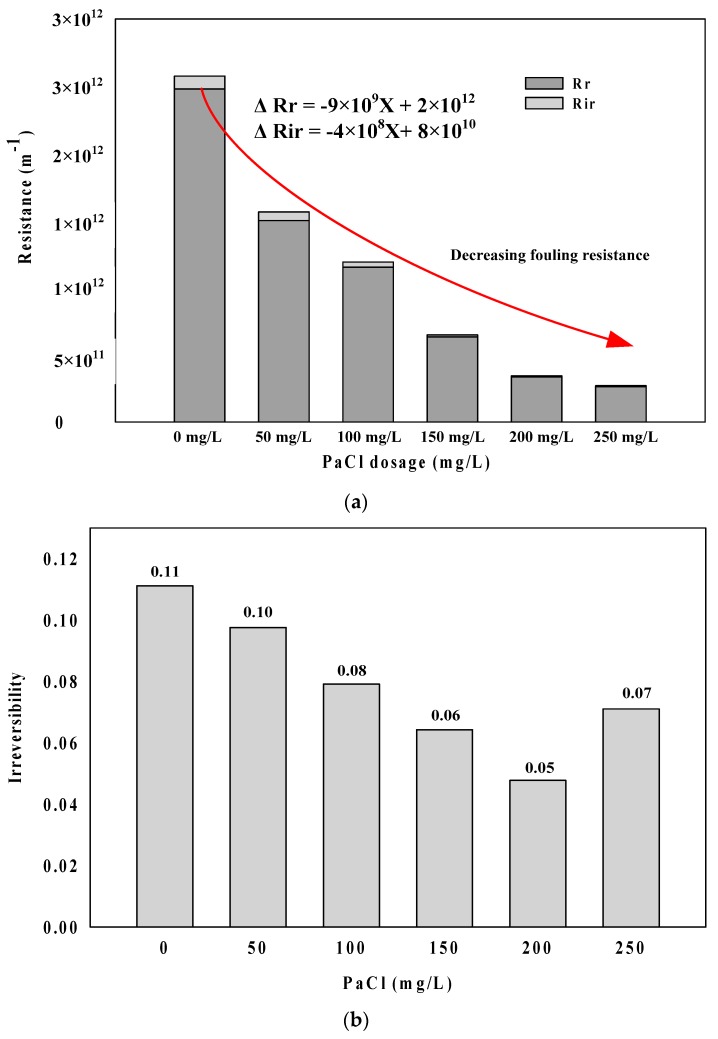
Results of reversible and irreversible analyses according to coagulant dosage. (**a**) R_r_ and R_ir_ filtration resistance with coagulant dosage rate; and, (**b**) Irreversibility analysis with coagulant dosage rate.

Figure 1 shows the changes in the ceramic membrane TMP (Transmembrane pressure) according to the coagulant dosage rate from 0 to 250 mg/L. It is observed that the rate of increase in TMP markedly decreases with increasing PaCl dosage. It has been reported algal cells, including similar substances, such as polysaccharides, proteins, lipids, and humus, and extracellular organic matter (EOM) cause the membrane fouling by algae [20]. Moreover, it is known that membrane fouling decreases with increasing the coagulant injection into these substances, because they are coagulated into larger aggregates [21]. The increase in TMP decreased rapidly with increasing the coagulant injection rate also in the results of these experiments. It is considered that algae particulate matter lowered the fouling resistance by coagulation. Figure 2a shows the reversible (R_r_) and irreversible (R_ir_) filtration resistance according to the coagulant injection rate. As shown in the figure, R_r_ and R_ir_ decreased with increasing the coagulant injection rate. The reversible fouling resistance (∆R_r_, −9 × 10^9^ X) showed a higher rate of decrease than the irreversible fouling resistance (∆R_ir_, −4 × 10^8^ X), according to the coagulant injection rate. In addition, Figure 2b shows the results of the irreversible analysis. Although the irreversibility decreases to 200 mg/L, it shows a tendency to increase from 250 mg/L. Wu et al. reported that membrane fouling was reduced within the reasonable coagulant dosage rate [22]. However, they reported that the excessive coagulant dosage increased the Zeta potential of raw water and then caused electrostatic repulsion to accelerate fouling by forming dense fouling layers. A similar tendency was found in this paper, and PaCl that was above the proper dosage (200 mg/L) dose increased irreversible fouling.

### 3.2. Mechanisms of Membrane Fouling Caused by Coagulation Dosage in Algal Rich Water

**Figure 3:**
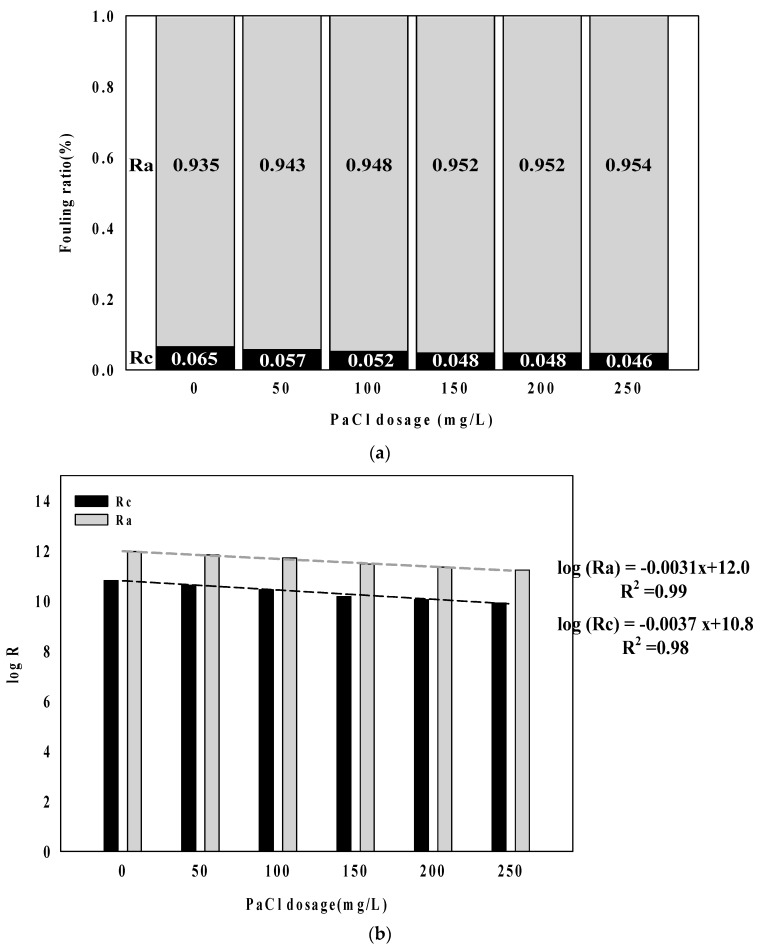
Analysis results of fouling characteristics with coagulant dosage. (**a**) Fouling ratio; and (**b**) log (R_a_ & R_c_).

Figure 2 shows R_a_ and R_c_ to analyze the membrane fouling mechanism with coagulant dosage in algal rich water. As shown in Figure 2a, the adsorption resistant (R_a_) showed a higher rate than cake resistance (R_c_) regarding filtration using membranes in algal rich water. The rate for R_a_ showed a tendency to gradually increase with an increasing coagulation injection rate. Figure 2b shows each injection rate condition in filtration resistance logarithmic values. log (R_a_) and log (R_c_) tended to decrease with increasing coagulant injection rate and log (∆R_c_) (−0.0031X) showed a higher decreasing trend than log (R_a_) (−0.0037X) (Figure 3b).

**Figure 4:**
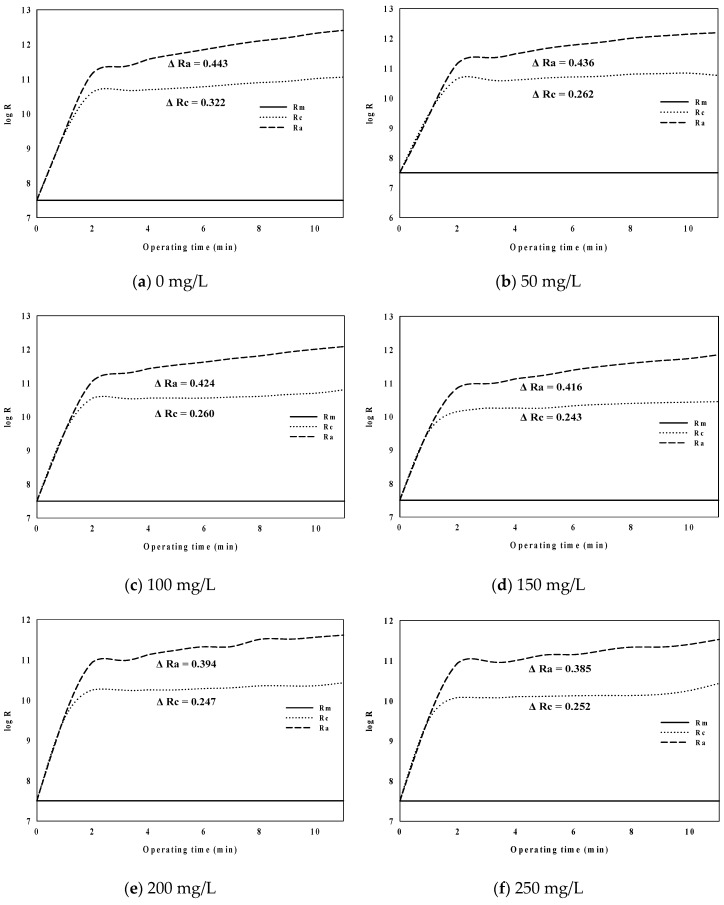
Behaviors of R_a_ and R_c_ with time.

Figure 4 shows the values of R_a_ and R_c_ with the passage of time according to coagulant dosage rate in the logarithmic values.

As shown in Figure 4, ∆log (R_a_) changes from 0.443 to 0.385 according to the coagulant injection rate from 0 mg/L to 250 mg/L and showed a tendency to decrease with an increasing injection rate. Although ∆log (R_a_) showed the overall decrease, it increased the above proper coagulant dosage (200 mg/L). It was confirmed that the above proper coagulant dosage in algal rich water was more closely related to the fouling by cake than to the fouling resistance by adsorption.

### 3.3. Specific Cake Resistance and Compressibility

**Figure 5:**
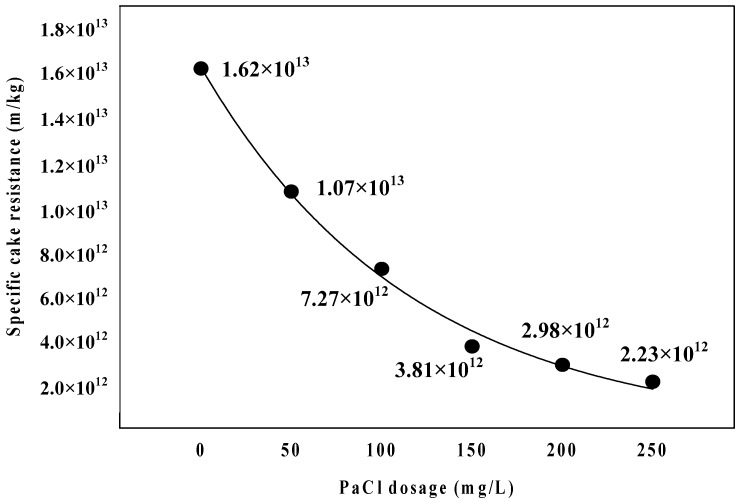
Comparison of cake specific cake resistance with coagulant dosage.

**Figure 6:**
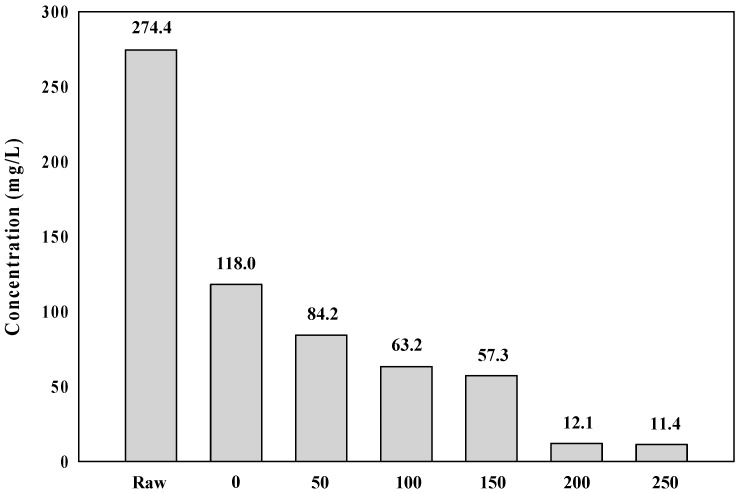
Polysaccharide concentration in feed and permeate water.

As shown in Figure 5, the specific cake resistance showed a tendency to decrease from 1.62 × 10^13^ to 2.23 × 10^12^ with an increasing PaCl dosage from 0 to 250 mg/L. According to Lee et al. [23], the floc structure had an effect on the specific cake resistance, and specific cake resistance increased with a decreasing floc structure, because it formed a more compact cake layer on the membrane surface. Moreover, Tabatabai et al. [24] reported that coagulation substantially reduced the fouling potential and compressibility of the AOM cake/gel layer by creating highly porous cakes. The membrane fouling resistance also decreased with an increasing PaCl dosage rate in this experiment, and it is considered that the specific cake resistance decreases because this floc forms a loose porous cake layer on the membrane surface. Figure 6 is the analysis results of polysaccharide in feed and permeate water. As the PaCl dosage rate increased, the polysaccharide concentration in the permeate water decreased. These results suggest that the small particles form large agglomerates by dosing coagulant, and it is thus removed by membrane. It was also confirmed that these agglomerates formed high porosity cake.

**Figure 7:**
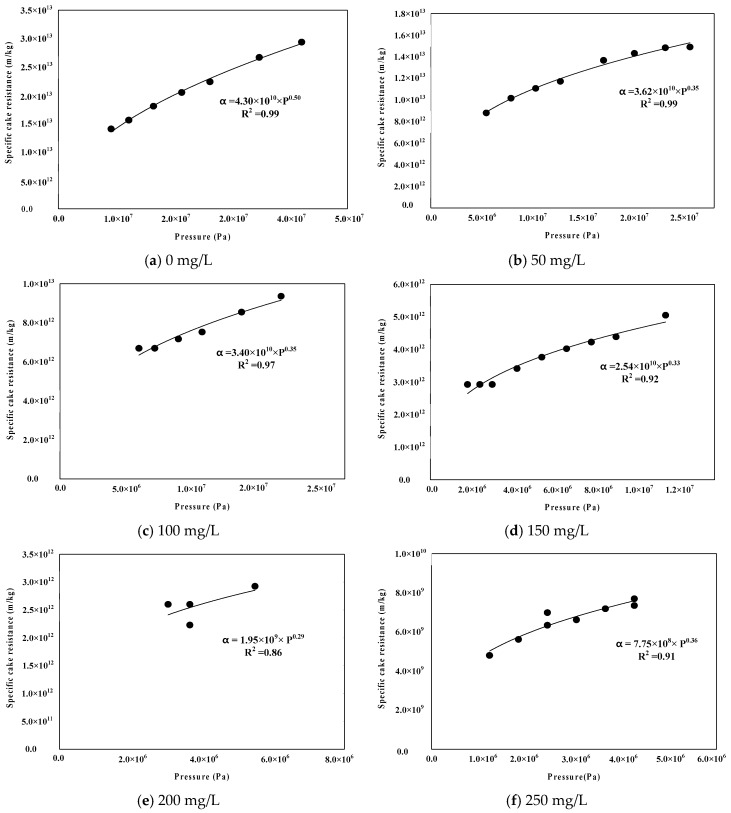
Compressibility index with Polyaluminum chloride (PaCl) dosage.

Figure 7 shows the results of the compaction coefficient of the cake layer through correlations with specific cake resistance, according to TMP. It can be said that the compressible cake index (n) is the value that is determined by the raw water characteristics because membranes with the same materials were used in the experiments to determine the compaction of the coefficient and the raw water conditions were changed. As shown in Figure 7, the initial specific cake resistance (α_0_) decreased continually from 4.30 × 10^10^ to 7.75 × 10^8^, according to PaCl dosage rate from 0 to 250 mg/L. On the other hand, the compressible cake index n decreased from 0.50 to 0.29, according to PaCl dosage rate from 0 to 200 mg/L, but it showed a higher value (0.36) at the dosage of 250 mg/L than that at the dosage of 200 mg/L. Table 3 summarizes the specific cake resistance and the compressible cake index. Specific cake resistance showed linear increase with increasing PaCl dosage, but compressible cake index tended to decrease within the proper dosage rate. However, it was confirmed that the dosing PaCl that above the proper dosage increased compressible cake index.

On the basis of these results, the increase in coagulant dosage within the proper dosage rate in membrane filtration according to coagulant dosage in algal rich water caused a decrease in the initial cake resistance and less impaction of the cake layer that was attached to the membrane layer. The initial cake resistance decreased, but the compressible cake index increased in injecting the coagulants above the proper injection amount. As mentioned in the introduction, it is considered that a decrease in the electrostatic repulsion between the membrane surface and algal particles formed a dense cake layer on the membrane surface.

## 4. Conclusions

This paper examined membrane fouling according to coagulant dosage using raw water that was sampled from the lake where algae occurred. This study was performed to understand the cake properties of algae particles that were attached to the membrane surface under various coagulation conditions. The following conclusions were drawn:

1. The rate of increase in TMP decreased with increasing PaCl dosage when filtering membranes in algal rich water. It was confirmed that PaCl dosage rate and TMP were important to each other.
2. The reversible and irreversible fouling resistance decreased with an increasing PaCl dosage rate. The irreversible rate increased above the optimal PaCl dosage (200 mg/L as PaCl).
3. Fouling resistance showed a tendency to decrease with an increasing PaCl dosage rate. As a form of membrane fouling, the adsorption resistant accounted for a higher proportion than cake resistance. In particular, cake resistance showed a higher decreasing trend than adsorption resistance. It is considered that an increase in the floc size according to coagulant played a causative role.
4. The specific cake resistance and compressible index were analyzed to examine the cake layer properties according to the PaCl dosage rate. As a result, the cake resistance decreased with an increasing PaCl dosage, but the compressible index showed a tendency to increase above the proper coagulant dosage. It is considered that the calculation of the proper coagulant dosage is an important factor controlling membrane fouling in membrane process of algal rich water.

## Figures and Tables

**Table 1:** Characteristics of the raw water.

Parameters		
Cell density (cells/mL)	Cyanobacteria	262,000
	Chlorophyta	116
	Diatom	318
	Other algae	-
	Total algae	262,434
Algal species	Dominant species	Microcystis
	Subdominant species	Synedra
pH		7.8–8.1
Suspended matter (mg/L)		AVG. 525.0
Turbidity (NTU)		AVG. 443.0

**Table 2:** Physical characteristics of membrane.

Characteristic Items	Properties
Membrane material	Silicon carbide
Effective filtration area (m^2^)	0.00652
Membrane type	Flat type
Pores if the MF membrane (µm)	0.1
Clean water permeability (LMH */bar)	5000 LMH/bar at 20 °C

* LMH = m^3^/m^2^·h.

**Table 3:** Specific cake resistance and compressible cake index with PaCl dosage.

Dosage	Specific Cake Resistance, α (m/kg)	Compressible Cake Index, n
0	1.62 × 10^13^	0.50
50	1.07 × 10^13^	0.35
100	7.27 × 10^12^	0.35
150	3.81 × 10^12^	0.33
200	2.98 × 10^12^	0.29
250	2.23 × 10^12^	0.36
